# Sarcoidosis versus Granulomatous and Lymphocytic Interstitial Lung Disease in Common Variable Immunodeficiency: A Comparative Review

**DOI:** 10.3390/biomedicines12071503

**Published:** 2024-07-06

**Authors:** Helena Buso, Claudia Discardi, Patrick Bez, Francesco Muscianisi, Jessica Ceccato, Cinzia Milito, Davide Firinu, Nicholas Landini, Mark G. Jones, Carla Felice, Marcello Rattazzi, Riccardo Scarpa, Francesco Cinetto

**Affiliations:** 1 Rare Diseases Referral Center, Internal Medicine 1, Department of Medicine (DIMED), AULSS2 Marca Trevigiana, Ca’ Foncello Hospital, University of Padova, 35124 Padova, Italycarla.felice@unipd.it (C.F.); marcello.rattazzi@unipd.it (M.R.); scarpa.riccardo@gmail.com (R.S.); francesco.cinetto@unipd.it (F.C.); 2 Haematology and Clinical Immunology Unit, Department of Medicine (DIMED), University of Padova, 35124 Padova, Italy; 3 Veneto Institute of Molecular Medicine (VIMM), 35131 Padova, Italy; 4 Department of Molecular Medicine, “Sapienza” University of Rome, 00161 Rome, Italy; 5 Department of Medical Sciences and Public Health, University of Cagliari, 09124 Cagliari, Italy; 6 Department of Radiological, Oncological and Pathological Sciences, Policlinico Umberto I Hospital, “Sapienza” University of Rome, 00161 Rome, Italy; 7 Clinical and Experimental Sciences, Faculty of Medicine, University of Southampton, Southampton SO16 YD, UK; mark.jones@soto.ac.uk; 8 Institute for Life Sciences, University of Southampton, Southampton SO17 1BJ, UK; 9 NIHR Southampton Biomedical Research Centre, University Hospital Southampton, Southampton SO16 6YD, UK

**Keywords:** granulomatous disease, sarcoidosis, granulomatous lymphocytic interstitial lung disease, common variable immunodeficiency

## Abstract

Sarcoidosis and Granulomatous and Lymphocytic Interstitial Lung Diseases (GLILD) are two rare entities primarily characterised by the development of Interstitial Lung Disease (ILD) in the context of systemic immune dysregulation. These two conditions partially share the immunological background and pathologic findings, with granuloma as the main common feature. In this narrative review, we performed a careful comparison between sarcoidosis and GLILD, with an overview of their main similarities and differences, starting from a clinical perspective and ending with a deeper look at the immunopathogenesis and possible target therapies. Sarcoidosis occurs in immunocompetent individuals, whereas GLILD occurs in patients affected by common variable immunodeficiency (CVID). Moreover, peculiar extrapulmonary manifestations and radiological and histological features may help distinguish the two diseases. Despite that, common pathogenetic pathways have been suggested and both these disorders can cause progressive impairment of lung function and variable systemic granulomatous and non-granulomatous complications, leading to significant morbidity, reduced quality of life, and survival. Due to the rarity of these conditions and the extreme clinical variability, there are still many open questions concerning their pathogenesis, natural history, and optimal management. However, if studied in parallel, these two entities might benefit from each other, leading to a better understanding of their pathogenesis and to more tailored treatment approaches.

## 1. Introduction

Sarcoidosis and Common Variable Immunodeficiency (CVID) are rare diseases, with an estimated prevalence between 2 and 150 over 100,000 of the general population for the former [1] and between 2 and 4 over 100,000 for the latter, affecting mainly adult patients [2].

While sarcoidosis is a systemic granulomatous disease typically occurring in immunocompetent adults, CVID is the most frequent symptomatic primary antibody deficiency diagnosed in adulthood, characterised by marked hypogammaglobulinemia, with consequent impairment in vaccine response, severe and recurrent infections, and immune dysregulation [3,4].

Granulomatous and Lymphocytic Interstitial Lung Diseases (GLILD) is one of the most severe non-infectious complications of CVID, reported in around 15–20% of cases [5], with some clinical and pathological features resembling a sarcoidosis-like picture.

Both sarcoidosis and GLILD are based on profound immune dysregulation, probably due to an impairment in antigen clearance, with granuloma formation as the main consequence.

GLILD is a quite recently defined entity that specifically occurs in the context of Inborn Errors of Immunity (IEI) and has been less extensively studied than sarcoidosis. Therefore, identifying GLILD can be challenging, especially when it is the first clinical manifestation of CVID [6]. Indeed, some studies from 1990s-early 2000 reported sarcoidosis as a possible comorbidity of CVID while recent studies reported CVID as an immune-mediated co-morbidity of sarcoidosis, confirming the difficulty of recognising GLILD in clinical practice and the importance of differential diagnosis [7].

We believe that a comprehensive comparison of these two diseases, starting from the clinical characteristics and moving through the analysis of the shared and different immunopathological substrates, could potentially provide new insights for a deeper understanding of their pathogenesis and the consequent definition of tailored follow-up and treatment protocols, to ensure the best patient management.

## 2. Methods

We conducted a narrative review, retrieving manuscripts published in the last twenty years. The search terms “Sarcoidosis AND Common Variable Immunodeficiency” OR “Common Variable Immunodeficiency AND granuloma” OR “Common Variable Immunodeficiency AND GLILD” OR “CVID-ILD” OR “Sarcoidosis” were used in PubMed. Written informed consent has been obtained from the patients to publish anonymised computed tomography (CT) and ^18^Fluorodeoxyglucose-Positron Emission Tomography/Computed Tomography (^18^FDG-PET/CT) images.

## 3. Clinical Manifestations

Sarcoidosis and GLILD are both systemic granulomatous diseases with preferential involvement of lungs and lymph nodes. They are characterized by a wide range of organ-specific and nonspecific manifestations, such as cough, exertional dyspnea, and constitutional symptoms [8,9,10,11]. Of note, in both disorders, a significant percentage of patients remain asymptomatic [12,13,14].

### 3.1. Extrapulmonary Involvement

Pulmonary symptoms and nodal enlargement are rarely isolated and often appear in the context of a more complex clinical picture with extrapulmonary involvement [10,15].

Neurological, cardiac, ocular, and renal involvement (including abnormalities of calcium metabolism) are possible extra-thoracic manifestations in sarcoidosis; all together occurring in almost 50% of patients [16,17,18]. On the contrary, such organ involvement is only anecdotally reported in GLILD [19,20,21,22]. Conversely, hepato-splenomegaly is reported in almost half of GLILD patients [8,11,23] and considerably less in sarcoidosis patients [24,25].

Skin involvement is consistently reported in both diseases, although the manifestations are heterogeneous and not pathognomonic [9,26], except for lupus pernio, which is characteristic of sarcoidosis).

### 3.2. Acute-Onset Clinical Pictures

Acute-onset clinical pictures with spontaneous remission are typical of sarcoidosis, particularly of the Lofgren syndrome (fever, bilateral hilar lymphadenopathy, erythema nodosum, bilateral ankle arthritis) and Heerfordt syndrome (fever, facial nerve palsy, uveitis, swelling of the parotid gland) [27]. These types of presentations are not reported in GLILD.

### 3.3. Infective, Autoimmune and Neoplastic Complications

The typical manifestations of CVID include recurrent and severe bacterial infections, especially of the respiratory tract, due to antibody deficiency, often associated with several immune-mediated diseases. In detail, autoimmune cytopenia (i.e., idiopathic thrombocytopenic purpura or autoimmune haemolytic anaemia) is the most frequent immune-mediated complication of CVID, occurring in around 10.4% of patients and in 59.6% of GLILD patients [28,29]. Polyclonal and clonal lymphocyte proliferation and non-infectious enteropathy are also typical of the complex CVID phenotype, as a consequence of immune dysregulation. As such, these manifestations should prompt the suspicion of an underlying primary immune defect. The importance of these associations is underpinned by the fact that available GLILD’s predictive models include autoimmune cytopenias and polyclonal lymphoproliferation (splenomegaly and lymphadenopathy) [14,28].

On the contrary, infections are reported in sarcoidosis as a consequence of immunosuppressive treatments. Autoimmunity (including autoimmune cytopenia) complicates sarcoidosis just occasionally and with a weaker association [30,31,32]. Thus, in the presence of such manifestations, immunodeficiency should necessarily be ruled out.

**Figure 1:**
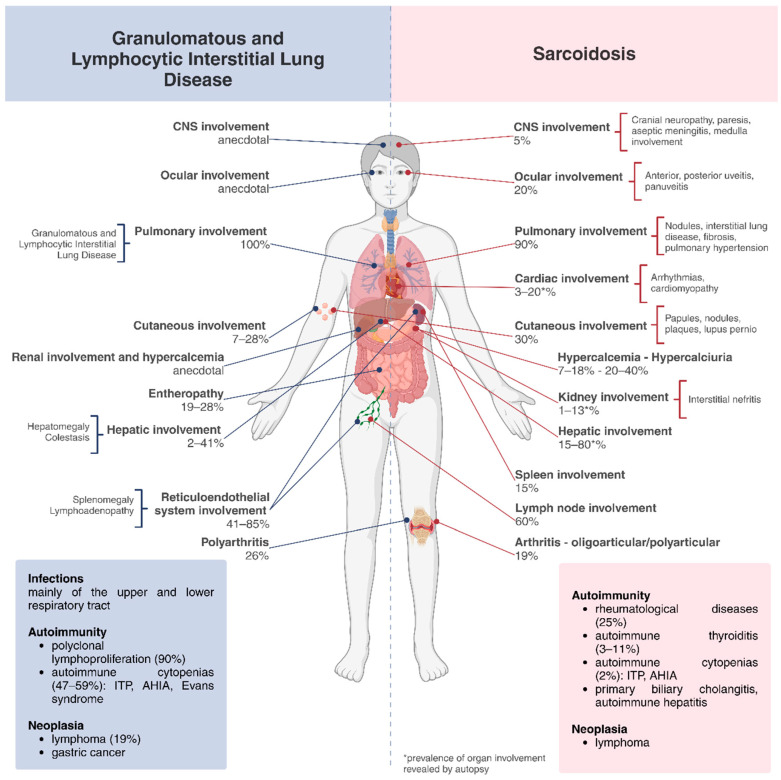
GLILD (Granulomatous and Lymphocytic Interstitial Lung Disease) vs. Sarcoidosis: granulomatous and non-granulomatous organ involvement with their relative percentage and disease complications. Abbreviations: ITP, immune thrombocytopenia; AHIA, autoimmune hemolytic anaemia. [8,10,11,15,18,19,24,25,26,28,30,31,32,41]. This figure has been created with BioRender.com.

Both diseases may be associated with cancer; CVID poses a greater risk of malignancy, particularly gastric cancer and non-Hodgkin lymphoma, than the general population [33] while sarcoidosis has been reported in association with lymphoma and any solid tumour (i.e., lung, skin, breast, thyroid, gastrointestinal, and genitourinary cancers) [32,34]. The association between sarcoidosis and solid neoplasia is documented particularly in the course of the first 2 years after the diagnosis, in the context of a so-called “sarcoid-like” reaction to malignancy [32]. In this condition, the granulomas may be found in the vicinity of the tumour, either in the cancerous organ itself, draining lymph nodes or adjacent to metastases [35]. Figure 1 summarize the clinical manifestations of sarcoidosis and GLILD.

### 3.4. Prognosis

Prognosis is quite different between GLILD and sarcoidosis. Sarcoidosis presents more often an indolent course, and it reaches a remission (also spontaneously) in about 30–50% of cases within 3 years since the diagnosis; the mortality rate is generally low, as it was reported to be about 4.32 per million population in an American cohort and 3.6 per million population in a French cohort [36,37]. However, sarcoidosis’ prognosis could be significantly affected by the occurrence of fibrosing interstitial lung disease and pulmonary hypertension or by extra-thoracic involvement, particularly if noble organs are severely impaired [16,38,39,40].

Instead, the majority of GLILD patients show a progressive disease course with a non-negligible mortality. Historically, Bates et al. described a mean survival of 10.9 years versus 28.8 years in CVID patients without a granulomatous disease [23]. Boursiquot et al. found a mortality rate of 13.5% in their GLILD cohort. GLILD mortality appears to be correlated with the deterioration of lung function or liver function and the appearance of non-granulomatous complications [8].

## 4. Diagnosis

### 4.1. Diagnostic Workup

Given the lack of definite diagnostic biomarkers, expert statements and clinical practice guidelines recommend that the diagnosis of sarcoidosis should be based on the three following criteria: (1) a “compatible” clinical–radiological presentation; (2) demonstration of a non-necrotising granulomatous inflammation in at least one tissue sample; (3) a reliable exclusion of alternative causes of granulomatous disease, including CVID-associated granulomatous and lymphocytic interstitial lung disease [42,43]. Unfortunately, clinical and radiological findings may vary widely, and non-necrotising granulomatous inflammation is nonspecific to sarcoidosis. Indeed, a number of occupational, infectious, inflammatory and neoplastic conditions can mimic sarcoidosis [44]. Therefore, the diagnosis of sarcoidosis is never definitive and should be open to reconsideration during the follow-up [43].

In 20–50% of cases, sarcoidosis is suspected because of some incidental findings on imaging tests performed for unrelated reasons (i.e., intrathoracic lymphadenopathy; abdominal lymphadenopathy; bone lesions). Constitutional and respiratory complaints are the most common symptoms driving the clinician to further investigations, but also symptoms or signs reliable to extrathoracic organ involvement may be the first manifestation [10,45,46,47,48,49].

Similarly, according to the 2017 UK Consensus statement, a GLILD diagnosis requires radiological, functional, microbiological, and histopathological assessment of the lung, with a careful exclusion of other defined conditions [5]. In CVID, the development of respiratory symptoms or incidental radiological or functional findings captured at regular screening may suggest a GLILD diagnosis. CVID patients, indeed, regularly undergo Pulmonary function tests (PFTs) and high-resolution computed tomography (HRCT scans), which may allow early detection of ILD in asymptomatic patients [50,51].

In both conditions, chest computed tomography (CT) (thin slice and continuous) without contrast medium is the main radiological technique in the diagnostic work-up [52] (see Section 4.2). Moreover, Pulmonary Function Test abnormalities, including both restrictive and/or obstructive patterns, support the diagnosis. In particular, a reduction of CO diffusion capacity is often present, reflecting parenchymal and interstitial lung damage [8,10,38].

In sarcoidosis, the tissue staining and culture to exclude both hematologic and solid tumours and the presence of specific microorganisms (e.g., mycobacteria), is strongly advised. Sampling of easily accessible superficial lesions (e.g., skin, superficial lymph nodes) should be preferred when possible [44,53,54]; when no obvious superficial lesions are detected, sampling of the lung or the hilar/mediastinal lymph node is usually carried out [10,46]. Confirmation with biopsy is not required in specific clinical settings: asymptomatic bilateral hilar lymphadenopathy, Loefgren syndrome, Heerfordt syndrome and lupus pernio, as these conditions are highly suggestive of sarcoidosis [42,43,45]. 

According to the aforementioned UK Consensus statement, a definite diagnosis of GLILD requires evidence of granuloma and/or lymphoid hyperplasia on surgical open lung or video-assisted thoracoscopic surgery (VATS) biopsy, and the sampling of the easily accessible lesions is not considered. However, due to the associated risks, the use of this technique is currently being discussed and transbronchial biopsy (TBB) and even extra-pulmonary biopsy have been used to confirm the diagnosis [52]. Transbronchial cryobiopsy, which has not been specifically investigated in GLILD but used in other ILDs, could be a less invasive technique, with a lower rate of complications while providing better diagnostic confidence compared to TBB [55,56,57]. The broncho–alveolar lavage fluid (BALF) examination may support the diagnosis by ruling out infections and by assessing the differential cell count with lymphocyte subpopulations (see Section 4.3) [43,53,58,59,60,61,62].

Of note, recent studies used the definition of “probable GLILD” for patients with a consistent clinical–radiologic picture but without histologic confirmation on lung biopsy, when alternative diagnoses had been reasonably excluded [28,58]. Differently from sarcoidosis, no strongly suggestive BALF immunophenotype findings are currently available in GLILD, even though the detection of CD21 low B cells could support the diagnosis [61]. However, microbiological investigation on BALF including bacteria, mycobacterial and fungal cultures should be performed. (see Section 4.3).

Moreover, over the last few years, several models integrating clinical–laboratory and radiologic features have been developed to support the diagnosis of GLILD without the need of histological confirmation. The following models were proposed: (a) Mannina et al.: hypersplenism, polyarthritis, and FVC less than 80% resulting in an area under the curve (AUC) of 0.9267 [14]; (b) Hartono et al. splenomegaly, autoimmune cytopenia (AITP or AIHA), low serum IgA (<13 mg/dL), and CD21low > 5% [63]; (c) Cinetto et al.: splenomegaly, autoimmune cytopenia (AITP or AIHA), CD21low percentage and DLCO with an AUC of 0.9861 [28]; (d) Cabanero-Navalon et al. proposed splenomegaly, lymphadenopathy, low CD8^+^ cells in peripheral blood and high Baumann’s CVID-ILD radiologic score with an AUC of 0.969 [64].

Lastly, genetic testing is part of the routine assessment for CVID patients but not for sarcoidosis, since patients with a CVID-like phenotype may present underlying genetic defects that might explain the reason for immune dysregulation, as well as represent an important clue for a tailored treatment approach in specific cases (e.g., CTLA-4 haploinsufficiency, LRBA deficiency) [65,66].

### 4.2. Imaging

#### 4.2.1. Chest X-ray

Chest X-ray is usually the first step in the diagnostic work-up of pulmonary diseases, and may be the first investigation in patients with sarcoidosis. More than 90% of patients show typical alterations that have been traditionally classified according to the Scadding Staging System in 5 stages: 0 (normal), I (bilateral hilar lymphadenopathy without pulmonary infiltrates), II (bilateral hilar lymphadenopathy with pulmonary infiltrates), III (pulmonary infiltrates without bilateral hilar lymphadenopathy), and IV (fibrosis) [10,67]. These stages are associated with different probabilities of spontaneous resolution, in particular, stage IV includes permanent damage and is associated with higher mortality [68].

As already mentioned, in the context of a typical clinical presentation (i.e., Lofgren’s syndrome), also the detection of lymphadenopathy at chest X-ray may support the diagnosis [61]. However, major issues of Chest X-rays are their low capacity to detect and characterise structural lung changes, as well as the poor inter-reader reproducibility, which limit their use in both diagnosis and monitoring of sarcoidosis [48]. On the other hand, In GLILD, chest X-ray alone has an even poorer diagnostic value, and CT should be the first step in the diagnostic workup [5,52].

#### 4.2.2. Chest Computed Tomography

Chest computed tomography (CT), which represents the gold standard imaging technique in lung disease assessment, is warranted in all cases of sarcoidosis with atypical clinical and/or radiographic findings and in patients with suspected interstitial lung involvement [69].

**Figure 2:**
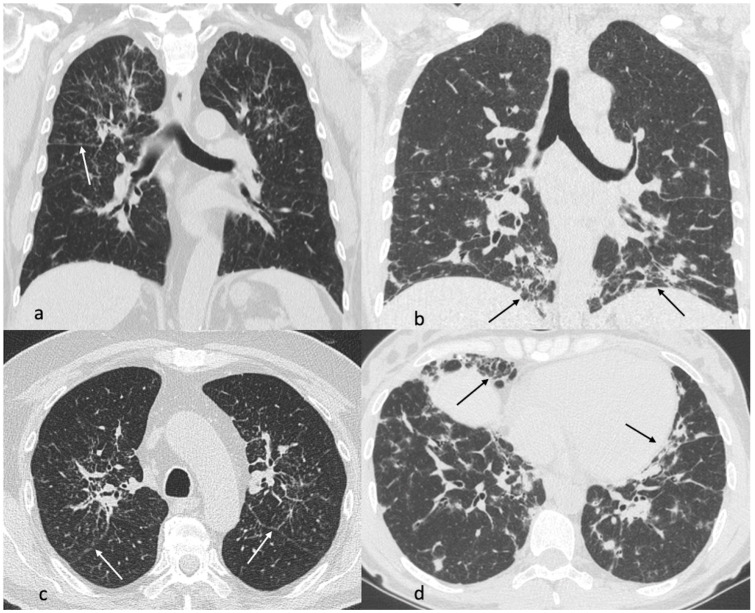
Male, 68 years old, sarcoidosis (**a**,**c**). Lung alterations predominate medium–upper fields with multiple perylimphatic nodules (white arrows, periscissural nodules) and perihilar fibrosis in upper lobes. Female, 38 years old, Common Variable Immunodeficency with GLILD (**b**,**d**). The disease is prevalent in lower fields, mainly sustained by consolidations and nodules. Reticulations (black arrows) with some signs of fibrosis are also appreciable.

Typical and atypical CT alterations have been described in both sarcoidosis and GLILD, with some common features and some differences (see Table 1, Figure 2) [70]. CT plays a key role in the diagnosis and differentiation between the two diseases, and the identification of peculiar CT features is important to limit the use of aggressive diagnostic tools, especially in GLILD patients [28].

In details, according to a recent study by Scarpa et al., the most common CT findings in GLILD, reported in more than two-thirds of patients, may be multiple non-perilymphatic small nodules (<10 mm), parenchymal scars/bands, consolidations, Ground Glass Opacities (GGO), low-grade bronchiectasis, and mediastinal enlarged lymph nodes [71].

Hence, despite the similarities with sarcoidosis, some differences may also be highlighted: first of all, while GLILD shows a prevalent involvement of lower fields, sarcoidosis usually affects the middle–upper lung fields [70,71,72,73]. Moreover, data on the distribution of lung nodules in GLILD are still few and partially conflicting [71], while in sarcoidosis, the nodules have a typical perilymphatic distribution [69]. Of note, consolidation and/or nodules with air bronchograms or halo signs are more frequently described in GLILD, while lymph node calcifications were described only in sarcoidosis [70,74]. Finally, bronchiectasis that is typically associated with GLILD is found only in fibrosis context, as traction bronchiectasis, in sarcoidosis [63,69].

In both diseases, different CT alterations may also suggest different stages of the disease, with a possible fibrotic end-stage evolution. Although there are no officially recognized scoring systems for assessing the degree of lung injury in GLILD and sarcoidosis, some scoring methods have been proposed. The Hartmann and Baumann scoring methods have been suggested for GLILD, while CT Activity Score (CTAS) has been proposed for sarcoidosis [75,76]. These scoring methods have yielded promising results, and, in the future, they may be validated to objectively define disease activity and determine the treatment response.

#### 4.2.3. Fluorodeoxyglucose (^18^FDG)-Positron Emission Tomography/Computed Tomography

**Figure 3:**
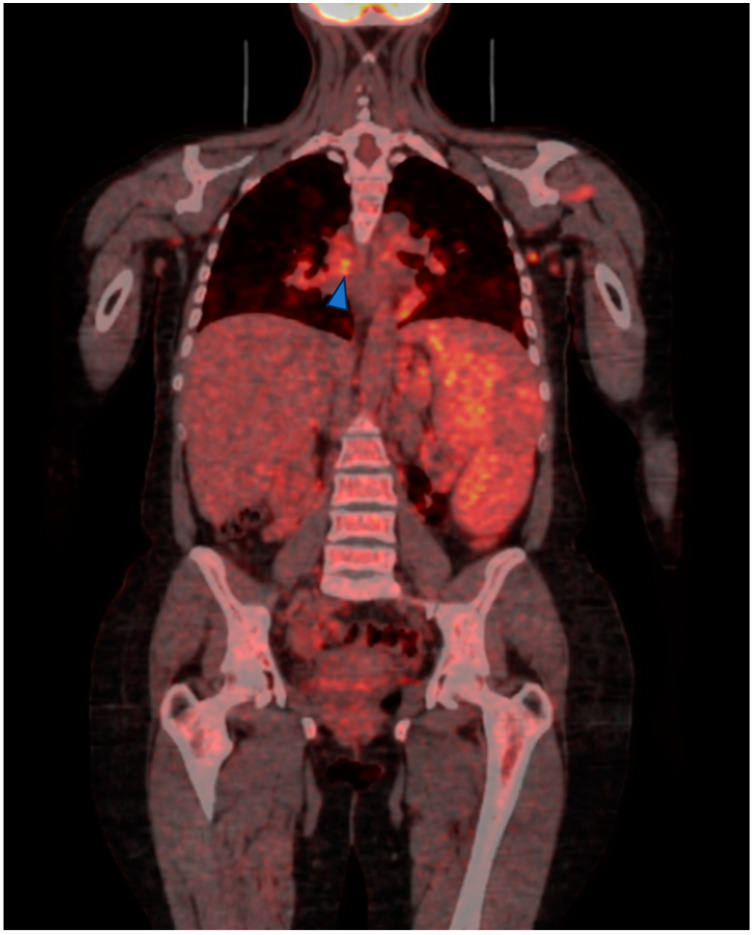
PEC-CT scan of a 34-year-old female affected by common variable immune deficiency complicated by GLILD. Disease activity is prevalent in lower lungs’ lobe, right hilar lymph node (arrow head); severely enlarged spleen has only mild FDG uptake.

**Figure 4:**
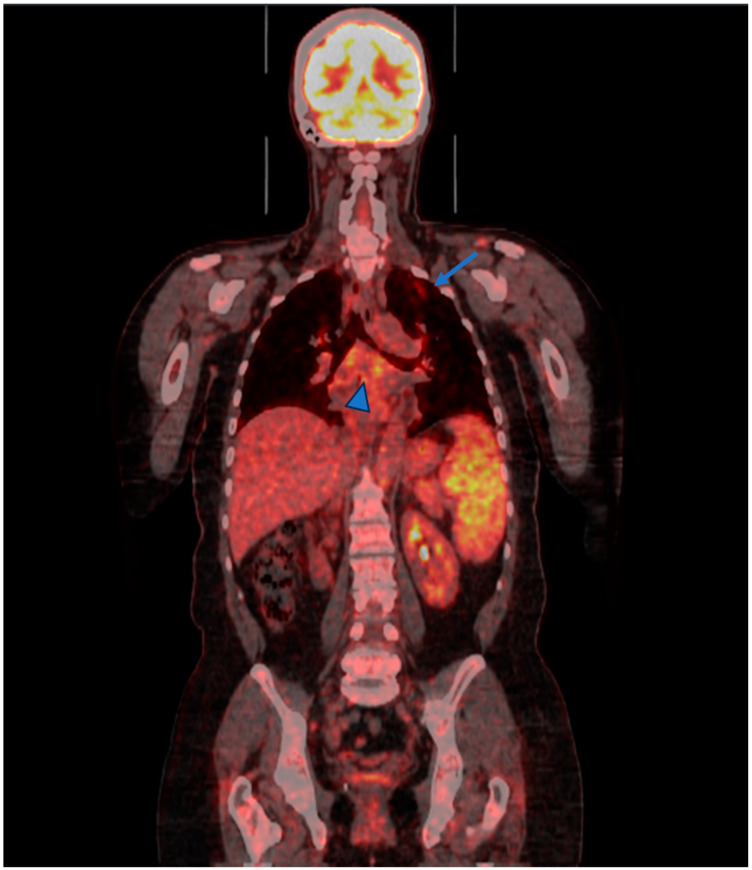
65 years old woman affected by sarcoidosis. Disease activity is prevalent in the left upper lobe (arrow) sub-carinal lymph node (arrow head) and spleen (mild enlargement, high and dishomogeneous FDG uptake).

Regarding other imaging techniques, the fluorodeoxyglucose (FDG)-positron emission tomography/computed tomography (PET/CT) scan may have a role in the assessment, differential diagnosis and monitoring of the response to treatment in both diseases (see Figure 3 and Figure 4). Indeed, the FDG PET-CT allows the evaluation of morphological and functional changes, especially providing information about the disease activity in both thoracic and extrathoracic involvement. However, its use should be limited to selected cases considering the radiation risk [77,78].

#### 4.2.4. Magnetic Resonance Imaging

While the use of magnetic resonance imaging (MRI) is well established for the detection of cardiac involvement in sarcoidosis [43] and it is already accepted in clinical practice in other diffuse lung diseases, such as cystic fibrosis [79,80], its role in the evaluation of ILDs is still controversial and remains a field of research [81]. In fact, CT has a superior morphological power that allows a better definition of lung and airway structural changes. However, MRI, without ionizing radiation exposure, could add functional and disease activity information that cannot be reached by CT, as already tested in patients with Primary Antibody Deficiencies [82,83]. Moreover, advanced morphologic MRI sequences seem promising also in the evaluation of ILD [84,85] and could support the already adopted MRI techniques, while dedicated low-field MRI scanner could boost the quality of lung MRI also ILD evaluation [86].

### 4.3. Bronchoalveolar Lavage Fluid (BALF) Immunophenotype

Lymphocytosis is a common finding in BALF of both GLILD and sarcoidosis. Friedmann et al. reported higher absolute lymphocyte count in GLILD patients compared to sarcoidosis [62].

Historically, in the bronchoalveolar lavage, a T lymphocytosis with a CD4/CD8 ratio greater than 3 to 4 has been considered an ancillary biomarker of acute sarcoidosis [59]. Nevertheless, several factors may impact on CD4/CD8 ratio, such as active smoking, obstructive pulmonary disease, more advanced stages of sarcoidosis, and treatment. A meta-analysis observed a sensibility of 0.7 and a specificity of 0.83 of the CD4/CD8 ratio for the diagnosis of sarcoidosis [87].

The analysis of BALF in GLILD patients has shown both a reduction and an elevation of CD4/CD8 T cells ratio [62,70,88]. Interestingly, a lower CD4/CD8 T cells ratio has been initially associated with a worse lung function (and consequently with poor prognosis) [88] while later it was correlated with a more pronounced lymphoid infiltration [89].

Regarding B cells, BALF of GLILD patients is characterised by an expansion of B lymphocytes, mainly CD21low T-bet high, and a reduction of switched memory B cells compared to sarcoidosis patients, that reflects the peripheral blood distribution. Higher levels of APRIL have also been detected in GLILD compared to healthy controls [62].

Instead, to our knowledge, B cells’ presence in BALF in sarcoidosis was not fairly investigated as it is considered mainly a T cell-mediated disease. However, recent studies focused on the role of B cells and their subsets, as well as their interaction with T helper cells, in BALF of sarcoidosis patients [90,91]. In detail, in one study by Bauer et al., a small proportion of antigen-experienced B cells was found in BALF [92]. Moreover, BAFF (B-cells activating factor) levels in BALF were correlated with increased BAFF levels in the serum, and both were associated with impaired respiratory function [93]. The identification of BALF biomarkers of sarcoidosis with a diagnostic and/or a prognostic value is a topic of great interest. For example, CD103^+^CD4^+^ T-cells in BALF of sarcoidosis patients are lower than in other ILD, while some chemokines able to recruit T-helper lymphocytes, such as CXCL9, CXCL10, and CXCL11, are increased. Moreover, increased numbers of Th17.1 cells, neutrophils, and NK cells in BALF appear to be correlated with a poorer prognosis and worse lung function. However, none of these biomarkers alone are specific enough [94].

### 4.4. Histopathology

The presence of non-necrotizing granulomas in the biopsies from different organs is a hallmark of both GLILD and sarcoidosis (see Table 2). However, in sarcoidosis granulomas typically have a uniform appearance, a perilymphatic distribution and a tendency to coalescence; in 6 to 35% of cases, focal caseating necrosis is present [95]. On the other hand, GLILD granulomas have various sizes, they do not show a lymphangitic distribution and they are usually non-necrotizing; lymphoid hyperplasia is frequently associated as the granuloma is either surrounded by a lymphocytic infiltrate or it is itself around the reactive follicle (see Table 2) [96].

Immunohistochemistry in sarcoidosis shows infiltration of CD4^+^ helper T cells inside the granuloma, whereas CD8^+^ cells are rarer [97]. Furthermore, a large number of CD20^+^CD79a^+^PAX5^+^ B-cells can be found around or inside the granuloma; CD138+ plasma-cells mainly producing IgA have been also described [98].

GLILD’s lung biopsies are more frequently characterised by the association of granuloma with lymphocytic interstitial pneumonia and follicular bronchiolitis [99]; organising pneumonia and pulmonary interstitial fibrosis can also be seen (see Table 2) [70,99]. However, none of these histologic findings are pathognomonic of GLILD [95]. In GLILD, lymph nodes are characterised by the presence of odd-shaped and ill-defined germinal centres, often with an abnormal polarisation and a CD8^+^ T-cells infiltration; plasma cells are severely reduced or absent and they reflect the peripheral antibody distribution, with IgM^+^ plasma cells being the most represented [100]. On the other hand, the evidence of a normal lymph node architecture with intact germinal centres and the presence of plasma cells (CD138^+^) supports a diagnosis of sarcoidosis rather than GLILD [100,101].

**Table 2:** Comparison of histological lung and lymph node findings in sarcoidosis and GLILD.

	Sarcoidosis	GLILD
**Granuloma**	Yes, perilymphatic distribution	Yes, but smaller undefined centrilobular/random distribution
**Pulmonary lymphoid hyperplasia (LIP, Follicular hyperplasia, lymphocytic infiltrates and nodular lymphoid hyperplasia)**	No	Yes
**Organizing pneumonia with an unusual dense lymphoid infiltrate**	No	Yes
**Fibrosis**	Frequent, concentric and lamellated around granulomas [102]	Yes
**CD4^+^ T cells**	Abundant, predominantly in the central zone of granuloma [102]	Predominant within lymphoid T cell infiltrate; surrounding B cells follicles [103]
**CD8^+^ T cells**	Predominantly in the outer cuff of the granuloma [102]	Scanty [103]
**Plasma cell (CD138^+^)**	Normal/augmented	Negative/reduced

For further details please refer to Section 4.4. Abbreviations: LIP: lymphoid interstitial pneumonia; CD: cluster of differentiation.

## 5. Pathogenesis

The pathogenesis of sarcoidosis and GLILD is still far from being completely understood, even if for sarcoidosis several steps forward have been made over the last few years. The granulomatous process is driven by alterations in both the innate and adaptive branches of the immune system. In CVID, in particular, the impairment in the immune response may justify both a higher risk of infections and a higher risk of translating external triggers into aberrant non-infectious inflammatory processes [104].

An aberrant response to chronic antigen exposure (e.g., Propionibacterium acnes, HHV8), has been hypothesised to be the first trigger in the disease development in both conditions [105,106,107]. In addition, exposure to several environmental factors, such as organic and inorganic dust, has been linked to the development of sarcoidosis [108,109]. The hypothesis of an aberrant immune response is further supported by the finding of JAK/STAT hyperactivation, possibly driven by interferons, in both sarcoidosis and CVID granulomas [96,110,111].

**Figure 5:**
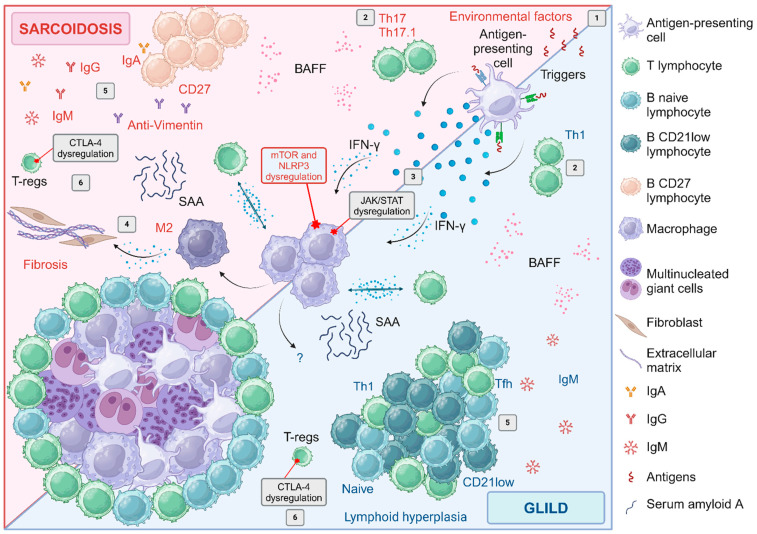
Similarities and differences in disease pathogenesis between sarcoidosis and GLILD (1) An aberrant response to chronic antigen exposure seems to be the first trigger in both diseases. In particular, exposure to several environmental factors has been linked to the development of sarcoidosis. (2) Antigens presenting cells (APC), activate the inflammatory response with T cell activation. The cytokine milieu influences the T cell polarisation toward Th17/Th17.1 response in sarcoidosis (IL-17 and IFN-γ production) and Th1 in GLILD (IFN-γ) activating the macrophages by JAK/STAT pathway. (3) Dysregulation of macrophage metabolism and activity (mTOR and NLRP3 pathways) is a key feature in sarcoidosis. Macrophages secrete different cytokines and inflammatory proteins (i.e., SAA) contributing to a Th17 cell shift and sustaining a proinflammatory environment with impairment in antigen clearance and consequent disease chronicisation. (4) In sarcoidosis classically activated macrophages (M1) are linked to granuloma formation, while alternatively activated macrophages (M2) are associated with the progression of the disease towards fibrosis. (5) A BAFF-driven lymphocytic hyperplasia with formation of tertiary lymphoid folliculi (and production of IgM) is typical of GLILD, with an increased proportion of follicular helper cells (CD4^+^, ICOS^+^, CD57^+^). Furthermore, expansion of naive-like CD21low B cells is reported in GLILD. Increased seric BAFF, which sustains B cells survival, is reported also in sarcoidosis. Differently from GLILD, in sarcoidosis B cells are of memory-like type with hyperproduction of immunoglobulins (serum hypergammaglobulinemia). Of note, anti-vimentin antibodies have been reported in a subgroup of sarcoidosis patients (HLA-DRB1*03). (6) Impairment of number and function of T-regs is reported in both diseases. In sarcoidosis, dysregulation of CTLA-4 expression has been demonstrated. Interestingly, CTLA-4 haploinsufficiency and LRBA deficiency (a gene associated with its regulation) are monogenic causes of CVID-like disorders associated with immune dysregulation. This figure has been created with biorender.com.

The peripheral monocytes of both CVID and sarcoidosis patients seem to have an intrinsically elevated tendency to fuse and form giant cells in comparison with monocytes from healthy controls [112,113]. Indeed, it is widely recognized that macrophages play a crucial role in the development and progression of sarcoidosis. Traditionally, classically activated macrophages (M1) are linked to granuloma formation, while alternatively activated macrophages (M2) are associated with the progression of the disease towards fibrosis, even if the M1/M2 paradigm is likely far too simplistic [114]. In this regard, several studies have described an alteration of mTOR pathways and a consequent dysregulation of macrophage metabolism as a key feature in sarcoidosis [115,116]. Moreover, the mechanistic target of rapamycin complex (mTORC), JAK/STAT and NLRP3 assessment by immunohistochemistry in tissue sampling from sarcoidosis patients revealed the potential simultaneous activation of all three pathways [117]. Furthermore, macrophages secrete different cytokines and inflammatory proteins (i.e., serum amyloid A). Notably, in sarcoidosis, macrophages contribute to a Th17 polarisation, sustaining a proinflammatory environment with impairment in antigen clearance and consequent disease chronicisation. On the other hand, only indirect evidence of mTOR pathways dysregulation is available in GLILD. Indeed, Sirolimus (an mTORC1 inhibitor), seems to be an effective treatment in GLILD [118]. All things considered, these suggest a similar underlying pathogenesis of granuloma in both disorders, as shown in Figure 5.

Moving from innate to adaptive immunity, different T cell subtypes have been implicated in the establishment and chronicisation of sarcoid granulomas [119]. As for sarcoidosis, also in GLILD a predominance of CD4^+^ T cells and a marked reduction of regulatory T cells (T-regs) in the lungs have been described [23,120]. In BALF of sarcoidosis patients, a polarisation towards Th17 and Th17.1 cells (able to produce both IL-17 and IFN-γ) has been described, being Th17.1 the predominant CD4^+^ population [119]. Friedmann et al. reported an increased proportion of T follicular helper (Tfh) in BALF of GLILD patients with polarisation toward Tfh1 and reduced Tfh17 compared to sarcoidosis [62]. Viallard et al. reported an increased proportion of Tfh (CD4^+^, ICOS^+^, CD57^+^) in biopsies from GLILD patients if compared with sarcoidosis patients [96]. In addition, a significant decrease of FoxP3^+^CD25^+^ T-regs among the memory CD4^+^ T cell compartment has been described, compared to sarcoidosis [62]. However, T-regs suppressive capacity and survival in peripheral blood and BALF of patients with sarcoidosis have been also shown to be impaired [121]. For instance, the dysregulated expression of co-inhibitory receptor cytotoxic T-lymphocyte antigen 4 (CTLA-4) in Th17 cells and T-regs in BALF and mediastinal lymph nodes of sarcoidosis patients possibly influences the local inflammation and is related to disease prognosis [122]. CTLA-4 is a critical immune checkpoint and target for cancer therapy. Notably, the use of anti-CTLA-4 monoclonal antibodies in cancer treatment is associated with the development of granulomatous disease. Moreover, genetic screening of patients with a CVID-like clinical phenotype and GLILD may reveal CTLA-4 haploinsufficiency or a deficiency of LRBA, a gene involved in CTLA-4 trafficking [66]. Reduced CTLA-4 and LRBA expression have also been described in CVID patients with autoimmunity, even in the absence of genetic findings [123]. Notably, restoring CTLA-4 function through Abatacept—a fusion protein of IgG1 with the extracellular domain of CTLA-4—has been proven effective in immune dysregulation in CVID [124,125] (section Treatment). This is a clear example of how a genetic diagnosis in GLILD patients might help clinical and therapeutic management, also highlighting one of the shared therapeutic targets between GLILD and sarcoidosis.

CD4^+^ T cells in sarcoidosis display hallmarks of exhaustion, such as poor response to T cell receptor (TCR), increased apoptosis and sustained PD-1 (programmed cell death protein 1) upregulation, probably as chronic antigen stimulation [126]. Recently, disrupted TCR signalling in HLA-DR^+^CD4^+^ T cells and CD8^+^CD57^+^ T cells in GLILD has been reported [127]. In this regard, Fraz et al. have shown an elevation of serum markers of T cell activation and exhaustion (TNFα, IFN-γ, sIL2R, sTIM-3), of pulmonary epithelial injury markers (CC16, SP-D), and of extracellular matrix remodelling markers in GLILD patients [128]. Similarly, also in sarcoidosis IFN-γ, produced by Th17.1 cells, and sIL2R were proposed as markers of disease activity [129,130] as well as CC16, SP-D, and MMP-7 as markers of interstitial lung damage [131,132,133].

Moving to B cells, their role in GLILD pathogenesis is well recognized. In fact, several studies have shown that in GLILD patients there is a peculiar distribution of B cells subtype in peripheral blood, with an expansion of CD21 low B cells in comparison with CVID patients without this complication [14,28]. This subset of B cells has already been associated with other autoimmune diseases such as Sjogren’s syndrome and systemic lupus erythematosus [94,95]. The finding of CD21 low B cells in the BALF of GLILD patients supports their pathogenic role [62]. Maglione et al. emphasised the role of B cells in CVID-ILD development, showing that a BAFF-driven B cell hyperplasia, reflected by serum IgM elevation, is correlated with ILD progression [134]. The key pathogenic role of B cells in GLILD is further supported by the observation that patients with X-linked agammaglobulinemia (XLA), a primary antibody deficiency characterised by an absence of B cells, seem to have a lower likelihood of developing ILD in comparison with CVID patients, in which there is an impairment of B cells function [135]. Moreover, according to a recent study by Verbsky et al., GLILD relapse after immunosuppressive treatment seems to be associated with an increase in the absolute value of B cells in the peripheral blood [136]. On the other hand, in sarcoidosis, only a small percentage of B cells is commonly found in BALF. These cells are CD27^+^ and IgD^−^, suggesting an antigen-experienced population. These cells have an expression profile resembling CD21low cells, albeit they are distinguished from classical T-bet^+^CD21^low^ cells by the expression of CD27 and higher levels of CXCR5 [92]. In both disorders, higher levels of BAFF compared to healthy donors have been repeatedly reported as a possible marker of disease activity [93,137,138]. Interestingly, polyclonal hypergammaglobulinemia is a typical finding in patients with active sarcoidosis. Furthermore, increased levels of immunoglobulins in BALF have been shown in sarcoidosis, suggesting local production of immunoglobulins in the lung [107,139].

Intriguingly, sarcoidosis has even been suggested to be an “autoimmune disorder” considering the evidence of in situ recognition of vimentin by both T- and B-cells in HLA-DRB1*03^+^ sarcoidosis patients, associated with the detection of anti-vimentin antibodies in BALF [140]. In consideration of the underlying hypogammaglobulinemia, the presence of autoantibodies is not described in GLILD patients.

Based on the pathogenesis several possible clinical biomarkers in blood and serum of sarcoidosis patients have been investigated (see Table 3 and Supplementary Table S1).

## 6. Therapy

Unfortunately, no good quality evidence-based treatment guidelines and management protocols are available for these two rare entities, even if the number of clinical trials performed for pulmonary sarcoidosis has increased over the last few years and at least consensus-based clinical practice guidelines are available [124,146].

### 6.1. Steroids

For both these diseases, treatment is required at the onset or worsening of respiratory symptoms, worsening of lung function and/or imaging progression, and noble organ involvement. Steroid monotherapy is usually considered a first-line therapy [5,146].

In sarcoidosis, ERS (European Respiratory Society) guidelines recommend starting with 20 mg of prednisone equivalent a day as in a recent randomised controlled trial high-dose prednisolone (40 mg) was not superior to a lower dose [147]. However, in clinical practice, the dosage might be higher, reaching sometimes 0.5–1 mg/kg a day especially when the central nervous system or the heart are involved; glucocorticoids are slowly tapered in 6 to 9 months [146,148].

For GLILD usually, a dose of prednisone equivalent to 40–60 mg is used, with a slow taper [124]. A recent multicentre retrospective study showed that a course of high-dose corticosteroids (considered as ≥0.3 mg/kg of prednisone equivalent) improved FVC and HRCT score in GLILD patients, achieving long-term remission in 42% of the 18 patients included [58]. In case of relapse, only 20% of them responded to another treatment course with steroids [58]. Similar efficacy of steroids was reported in another study [149].

### 6.2. Disease-Modifying Antirheumatic Drugs (DMARDs)

According to 2021 ERS guidelines, second-line treatment with immunosuppressive drugs should be initiated in sarcoidosis patients (i) requiring high doses of glucocorticoid to control their symptoms and who are unable to taper; (ii) suffering from unacceptable side effects of glucocorticoid treatment. Current options in this context include methotrexate (MTX), azathioprine, leflunomide, and mycophenolate mofetil. MTX is a folic acid antagonist with anti-inflammatory and immunosuppressive effects while azathioprine is a purine metabolism inhibitor [146]. Both MTX (a folic acid antagonist) and azathioprine (a purine metabolism inhibitor) have similar side effects, primarily myelosuppression, infections and gastrointestinal distress. MTX, but not azathioprine, is potentially teratogenic, which must be considered when treating young adults. Mycophenolate mofetil is a purine nucleotide synthesis inhibitor and was tested in a small study showing a sufficient steroid-sparing effect for patients with neurosarcoidosis [150].

Mycophenolate mofetil (MMF) in combination with steroids improved lung function in GLILD patients, allowing tapering of steroid therapy without relapse [149]. Evidence on the use of DMARDs in GLILD is limited and mainly reported in the context of combination therapies (azathioprine or mycophenolate) [136].

### 6.3. Target Therapies

Nowadays, the use of target biological therapies/small molecule inhibitors, based on the previously discussed pathogenetic pathways, represents the main novelty and, at least for sarcoidosis, new evidence is available or upcoming from clinical trials.

Anti-TNF treatment already represents a cornerstone for sarcoidosis treatment. As TNFα is an important mediator of T-helper lymphocytes and macrophages interaction in the granuloma formation (see pathogenesis) its inhibition became an important strategy in severe and relapsing/refractory disease [146,151,152]. Mainly infliximab, but also adalimumab, was shown to be effective in the treatment of pulmonary manifestations in 70–79% of cases [151,152] but also extra-pulmonary sarcoidosis in 74.5% of cases [149]; in particular, they were beneficial in the nervous system [153], cardiac [154], cutaneous [155], and ocular involvement [156], allowing steroids tapering. However, disease relapses occurred when the anti-TNF administration was delayed or suspended. Infliximab use has also been reported in granulomatous disease associated with CVID, but mainly for extrapulmonary involvement [157]. Also, anti-IL6 receptors and JAK inhibitors have shown good efficacy in sarcoidosis [158,159].

Recently, the treatment with the mTOR inhibitor sirolimus was proven effective in cutaneous sarcoidosis [160], showing promising results also in pulmonary disease [161]. As already mentioned, the use of Sirolimus has also been reported in GLILD, supporting the possible role of the mTOR pathway in the pathogenesis [118].

On the other hand, starting from the rationale of the role of B cells in the pathogenesis, rituximab, an anti-CD20 drug, either alone [162,163] or in combination therapy (i.e., azathioprine or MMF), is one of the most promising therapies for GLILD [136,164]. Interestingly, rituximab has been reported effective also in cases of refractory sarcoidosis patients [165].

Moreover, as discussed above, the identification of underlying gene defects in CVID patients may also support a tailored approach for GLILD. For example, Abatacept was successfully used in patients with CTLA-4 haploinsufficiency and in patients with LRBA mutation with granulomatous manifestations [124,125].

Of note, on the basis of the above-mentioned evidence regarding disease pathogenesis, abatacept is currently under investigation in a clinical trial for sarcoidosis [166]. All these further support the shared immunologic background of these two rare diseases.

Finally, belimumab, an anti-BAFF, might be a promising alternative treatment approach, considering the above-mentioned role of BAFF in the pathogenesis of both diseases.

## 7. Conclusions and Future Directions

As recapitulated in this narrative review, sarcoidosis and GLILD are two rare distinctive entities that share key features such as the prevalent interstitial lung involvement, the development of systemic complications and granuloma formation. On the other hand, the prevalence and type of extrapulmonary manifestations, and laboratory, radiological, and histological findings may distinguish the two clinical entities. Furthermore, sarcoidosis occurs in the context of immunocompetent individuals, whereas GLILD occurs in immunodeficient patients. Nonetheless, emerging evidence suggests the existence of shared immunopathogenic pathways.

Due to the low number of patients affected, high-quality evidence is rarely obtained and, as a consequence, standardisation of management protocol is challenging in both disorders. This review emphasises the significance of investigating these two entities in parallel, with a potential for mutual benefit at different levels. This could indeed facilitate a deeper understanding of the underlying disease mechanisms while paving the way for more targeted treatment approaches. On this basis, we believe that further studies may focus for example on the role of B cells and CVID-related genes in sarcoidosis and on the role of macrophage metabolism and innate immunity in GLILD, possibly offering new insights for a tailored approach.

## Figures and Tables

**Table 1:** Comparison of common CT findings in sarcoidosis and GLILD.

	Sarcoidosis	GLILD
**Common radiological lesions and distribution**	Predominantly upper lobe perilymphatic micronodular opacities with broncho–vascular distribution	Lower lobe nodular opacities with random or perilymphatic distribution
**Bronchiectasis**	Infrequent, mainly traction bronchiectasis	Common findings (in patients with recurrent infections)
**Mediastinal hilar adenopathy**	May be present	Common finding

Abbreviations: CT: chest tomography; GLILD: granulomatous lymphocytic interstitial lung disease.

**Table 3:** Comparison of disease and activity biomarkers in sarcoidosis and GLILD.

	Sarcoidosis	GLILD	Comment
**Gamma-globulins**	Polyclonal hypergammaglobulinemia	Hypogammaglobulinemia (IgG/IgA)	Produced by lung B cells in sarcoidosis; intrinsic B cell impairment in CVID
**Normal or elevated IgM**	Normal levels. No difference compared to healthy controls [141]	Increased levels associated with progressive disease [134]	Marker of B cell hyperplasia in the lung
**ACE in serum**	Increased [142]	Increased [143]	Produced by granuloma; established marker of activity in sarcoidosis
**BAFF in serum**	Increased [137]	Increased [134]	Produced by macrophages; marker of B cell hyperplasia
**Calcium-fosfate homeostasis alteration**	Increased vitamin D 1,25 (OH), Hypercalciuria, Hypercalcemia, inhibition of PTH	Not studied	Granuloma produces the active form of vit. D 1,25 (OH)
**SAA in serum**	Increased [144] but not specific of sarcoidosis	Not studied	Produced by macrophages; associated with chronicization and fibrosis
**sIL2R (sCD25) in serum**	Increased [145]	Increased [128]	Produced by activated lymphocytes; marker of disease activity
**CD21low in peripheral blood**	Not studied	Increased [63]	Chronically activated/ exhausted B cells

Abbreviations: ACE: angiotensin-converting enzyme; SAA: serum amyloid A; BAFF: B-cell activating factor; sIL2R: soluble receptor of interleuchin 2; CD: cluster of differentiation.

## Data Availability

Data were obtained from the Sarcoidosis registry (STUDY NUMBER 771/CE Marca) and GLILD.it study (STUDY NUMBER 1342/CE Marca). Both studies are coordinated by the Rare Immunologic and Respiratory Diseases Referral Center (Internal Medicine 1, Ca’ Foncello Hospital, Treviso, Department of Medicine—DIMED, University of Padova, Italy).

## Supplementary Materials

The following supporting information can be downloaded at: https://www.mdpi.com/article/10.3390/biomedicines12071503/s1, Table S1: Markers under investigation in sarcoidosis and GLILD. References [62,128,133,134,143,145,167,168,169,170] are cited in the Supplementary Materials.
