# Supplementary material for: Sarcoidosis versus Granulomatous and Lymphocytic Interstitial Lung Disease in Common Variable Immunodeficiency: A Comparative Review
Source: Biomedicines. 2024 Jul 6;12(7):1503. doi: 10.3390/biomedicines12071503 (PMC11275071; doi:10.3390/biomedicines12071503)
Supplement: Supplementary file 1 [file biomedicines-12-01503-s001.zip › biomedicines-3025498-supplementary.pdf]

	Sarcoidosis	GLILD	Comment
**sIL2R/WBC ratio in blood**	Increased compared to HD [143]	Increased [143]	May be a marker of progressive granulomatous disease in GLILD; higher than in sarcoidosis
**Chitotriosidase in serum**	Increased [167]	Not studied	Marker associated with activity and severity
**Neopterin in serum**	Increased	Not studied	Distinguish sarcoidosis vs healthy donor
**TNFα, IFNγ, sIL2R, sTIM-3 in serum**	Not studied	Increased compared to other CVID patients including those with other complications [128]	Marker of T-cell exhaustion
**SP-D and CC16 in serum**	Not studied	Increased compared to infection-only [128]	Marker of lung injury
**MMP-7 in serum**	Increased [133]	Increased compared to other CVID patients including those with other complications [128	Matrix remodelling
**YKL-40**	Increased [145]	Increased compared to infection-only [128]	Matrix remodelling
**CXCL9, 10, 11 in serum**	Increased [168][169]	Elevated in complicated CVID [170]	IFN-gamma related genes (CXCL9 associated with systemic involvement; CXCL10 with severity of lung involvement
**sCD163, sCD206 in serum**	Not studied	Increased [143]	Marker of Macrophage activation
**serum sCD28, sCD6, sCD83, IL-10, and LAMP3 in serum**	Not studied	Increased [170]	Discovered through proteomic approach
**CD4/CD8 T cell ratio in BALF**	Typically increased	Variable	
**BAFF in BALF**	Not studied, probably high	Increased [134]	Survival factor for B cells
**APRIL in BALF**	Not specifically investigated	Increased	Survival factor for B cells
**CXCL10 IN BALF**	Increased [62].	Increased [62].	Chemokine attracting CXCR3 positive Th1 cells and $CD21^{low}$T-bet^{hi} B cells
**MMP-7 in BALF**	Increased, correlated negatively with lung function (Isshiki)	Not studied	Matrix remodelling

The identification of BALF biomarkers of sarcoidosis with a diagnostic and/or a prognostic value is a topic of great interest. For example, CD103+CD4+ T-cells in BALF of sarcoidosis patients are lower than in other ILD, while some chemokines able to recruit T-helper lymphocytes, as CXCL9, CXCL10, CXCL11 are increased. Moreover, increased numbers of Th17.1 cells, neutrophils, and NK cells in BALF appear to be correlated with a poorer prognosis and a worse lung function. However, none of these biomarkers alone is specific enough [94].
